# Interface-designed Membranes with Shape-controlled Patterns for High-performance Polymer Electrolyte Membrane Fuel Cells

**DOI:** 10.1038/srep16394

**Published:** 2015-11-10

**Authors:** Yukwon Jeon, Dong Jun Kim, Jong Kwan Koh, Yunseong Ji, Jong Hak Kim, Yong-Gun Shul

**Affiliations:** 1 Department of Chemical and Biomolecular Engineering, Yonsei University, 50 Yonsei-ro, Seodaemun-gu, Seoul 120-749, Korea

## Abstract

Polymer electrolyte membrane fuel cell is a promising zero-emission power generator for stationary/automotive applications. However, key issues, such as performance and costs, are still remained for an economical commercialization. Here, we fabricated a high-performance membrane electrode assembly (MEA) using an interfacial design based on well-arrayed micro-patterned membranes including circles, squares and hexagons with different sizes, which are produced by a facile elastomeric mold method. The best MEA performance is achieved using patterned Nafion membrane with a circle 2 μm in size, which exhibited a very high power density of 1906 mW/cm^2^ at 75 °C and Pt loading of 0.4 mg/cm^2^ with 73% improvement compared to the commercial membrane. The improved performance are attributed to the decreased MEA resistances and increased surface area for higher Pt utilization of over 80%. From these enhanced properties, it is possible to operate at lower Pt loading of 0.2 mg/cm^2^ with an outstanding performance of 1555 mW/cm^2^ and even at air/low humidity operations.

In order to achieve a sustainable future, polymer electrolyte membrane fuel cells (PEMFCs) have been proposed as a promising alternative due to their high efficiency and environmental compatibility for potential applications in non-polluting transportation, stationary and portable electronics^1^,^2^,^3^. Although PEMFC technology has provided high performance competitive with those of alternative technologies, several challenges remain for commercialization such as the high cost, reliability and performance of membrane electrode assemblies (MEAs)^4^,^5^,^6^,^7^. To address these problems, the most important issue is finding a way to improve the interfacial contact between the electrode and membrane in the MEAs, as the interface properties are the primary hindrance impeding and retarding the commercial application of PEMFCs.

Membrane patterning is a favorable method to control the desired active surface area and conducting pathway in order to achieve enhanced electrochemical properties. Patterning techniques have been widely studied in various fields, especially in the research of electronic devices such as batteries and solar cells, as a means of boosting the efficiency with a larger active area and periodic photonic structures^8^,^9^,^10^,^11^. In fuel cell applications, the use of membranes with a patterned structure provides a better interfacial contact between the electrode and membrane^12^,^13^,^14^,^15^,^16^,^17^,^18^,^19^. Some research groups have been worked on the design of the structural PEMs prepared by various methods such as thermal imprint lithography^12^,^13^, electron beam^14^ and a plasma etching^15^, imprinting with a patterned silicon molds under high pressure/temperature (over 150 °C)^16^,^17^ and templates such as ZnO nanorods and porous polytetrafluoro-ethylene (PTFE)^18^,^19^. However, these fabrication processes are seen to be rather expensive and complicated to use for mass production and the high temperature could have an influence to the polymer membranes.

Effective MEA design can facilitate the formation of effective electrons, protons, reactants, and products pathways with high current-carrying capacity, and results in higher efficiency with minimized electrical losses^13^,^14^,^15^,^16^,^17^,^18^,^19^. Patterning also can result in an increased contact area with the catalyst nanoparticles by a high metal dispersion, leading to a faster catalytic reaction rate where an enhanced performance is caused mostly by the cathodic concentration overvoltage^20^,^21^,^22^. The metal dispersion by available surface area also directly affects the utilization of exposed metal atoms as the catalytic effects in MEAs are generated by a surface phenomenon^23^,^24^,^25^. Pt utilization in commercial Pt/C in PEMFC occurs in about 35–50% when the particle size ranges from 2–4 nm^26^,^27^,^28^. To achieve high Pt utilization, it is generally important to provide a good interaction between the Pt on a support and ionomer, resulting in a triple-phase contact that permits protonic access while encouraging diffusion of the gaseous species. The effective design of MEA can improve this metal utilization by creating better dispersion through the higher electrochemical surface area to obtain high PEMFC performance. The size and shape of the patterns are considered as the most important factors that are directly related to the membrane surface area. Aizawa *et al.* reported the effect of the height of a pillar at constant width and pitch, which outperformed the flat membrane even at low humidity^12^,^13^. Changing the shape of the pattern can also increase this interfacial area by effectively control the MEA design, which would provide the possibility for a reduction in the Pt-based catalyst amount. However, no systematic research was been performed using different pattern shapes and sizes.

Meanwhile, in order to compete with the current technologies, US Department of Energy (DOE) in its Fuel Cells Program Plan established a severe technical MEA performance target of over 1000 mW/cm2 by 2020 at specific operation conditions such as H_2_/Air and low humidity^29^. The DOE set up also a target in transportation applications for the reduction of total Pt group metal loading to 0.125 mg/cm^2^, from a current Pt loading of approximately 0.4–1.0 mg/cm^2^ to reduce the price of the fuel cell vehicles^29^,^30^,^31^. Therefore, to achieve this severe targets, we prepared a large-area platform through well-arrayed micropatterns with various sizes and shapes in order to investigate the influence of the interface structural effects, for the first time in detail on the electrochemical properties in relation to the PEMFC performance. A variety of patterned structures, i.e. circles, squares, and hexagons with different sizes, were generated using an elastomer mold of poly(dimethyl siloxane) (PDMS). This methodology is fast, facile, versatile and effective for producing a well-defined membrane structure and does not require high-temperature pressing, thus preventing chemical degradation of the membrane. The patterned Nafion membrane exhibited an exceptional performance of 1906 mW/cm^2^ at Pt loading of 0.4 mg/cm^2^ with a Pt utilization of over 80%, which shows 73% higher value in performance than the commercial membrane. These results indicate that control of the interface structure by patterning technique is important and potential feature to obtain high PEMFC performances at lower Pt loading of 0.2 mg/cm^2^ and at air/low humidity operation.

## Results

### Preparation and characterization of patterned membranes

**Figure 1:**
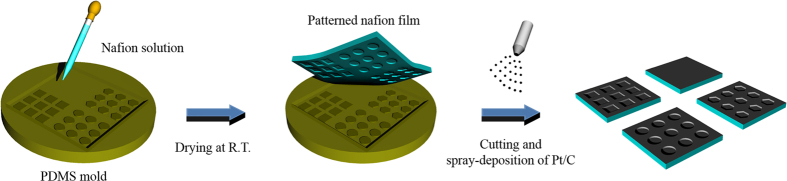
Preparation of the patterned membranes using a PDMS mold and catalyst deposition.

The patterned membrane was fabricated via a simple solution-derived procedure as illustrated in Fig. 1. First, the patterned PDMS mold was prepared using a 0.5-mm-thick silicon master with circular, hexagonal, and square intaglio patterns. A solution of PDMS monomer and curing agent at a 10:1 wt. ratio was poured onto the silicon master and cured at 70 °C for 1 h. A specific amount of commercially-available 5% Nafion solution was then cast onto the patterned PDMS mold to generate the patterned membrane. For fair comparison of MEA performance, the thickness of each membrane was controlled at approximately 47 ± 2 μm and the height of all patterns was fixed at approximately 1 μm^32^. After drying in a closed system at room temperature for 12 h, the Nafion membrane was slowly detached from the mold. The PDMS mold was reusable for generating a patterned membrane, indicating cost, environmental and process effectiveness. The patterned membrane was cut into each of the patterns, and the Pt/C was deposited onto the membrane with a direct spray method.

**Figure 2:**
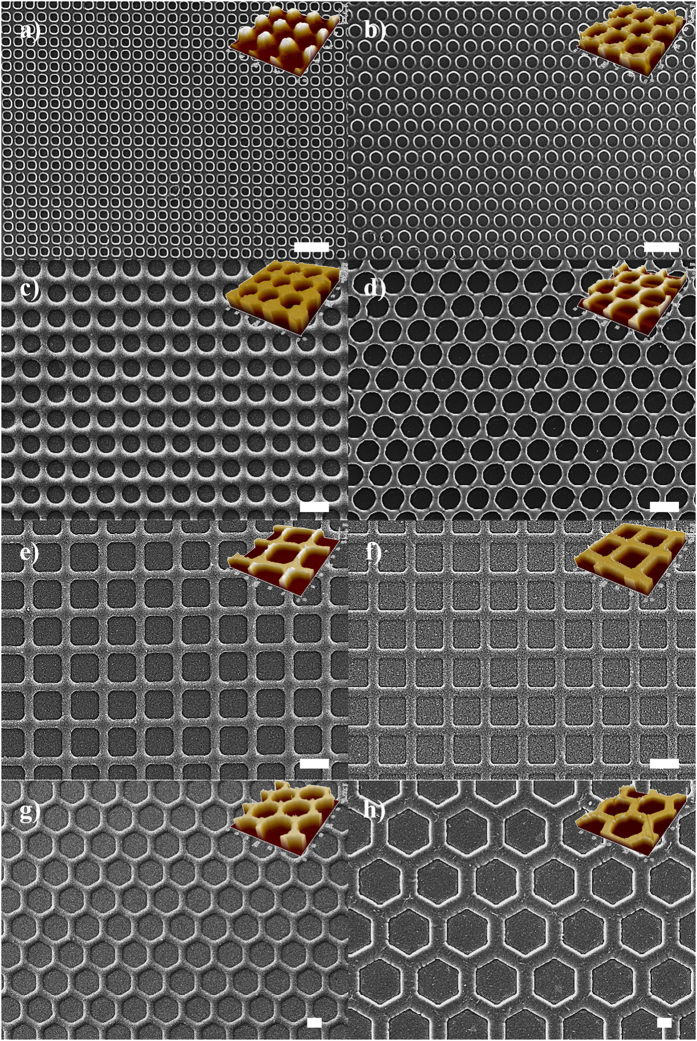
SEM images and 3-dimensional AFM images (insets) of patterned membranes (scale bars represent 10 μm). (a) Cir1, (b) Cir2, (c) Cir3, (d) Cir4, (e) Squ1, (f) Squ2, (g) Hex1, and (h) Hex2.

The patterned membranes were cut into eight different samples with different sizes of four circles, two squares, and two hexagons. The size of each pattern and their respective specific areas are listed in Table 1. The surface morphologies of membranes were characterized using a scanning electron microscope (SEM) as shown in Fig. 2. The structure and size of each pattern was varied from circles with a diameter of 2 μm (Cir1), 3 μm (Cir2), 5.5 μm (Cir3), or 7 μm (Cir4); squares with sides of 15 μm (Squ1) or 25 μm (Squ2); and hexagons with distance between two sides of 9 μm (Hex1) or 17 μm (Hex2). The specific membrane surface areas in Table 1 were calculated by multiplying the number of patterns in the specific area (1 cm^2^) and the side area of each pattern; thus, the plain membrane without a pattern had an area value of 1. The specific surface areas of the patterned membranes were always greater than that of the plain membrane, from a minimum 6.4% to a maximum 64.8%. With only surface SEM images, it was difficult to determine whether the patterns were a relief or intaglio pattern, but the 3D AFM images (insets of Fig. 2) revealed clear intaglio patterns for all surfaces, including that of the silicon master. When a monochromic light was exposed incident to the surface using a commercial laser pointer as a light source, the specific diffraction pattern appeared, indicating periodic alignment of the patterns, as shown in Fig. S1.

**Figure 3:**
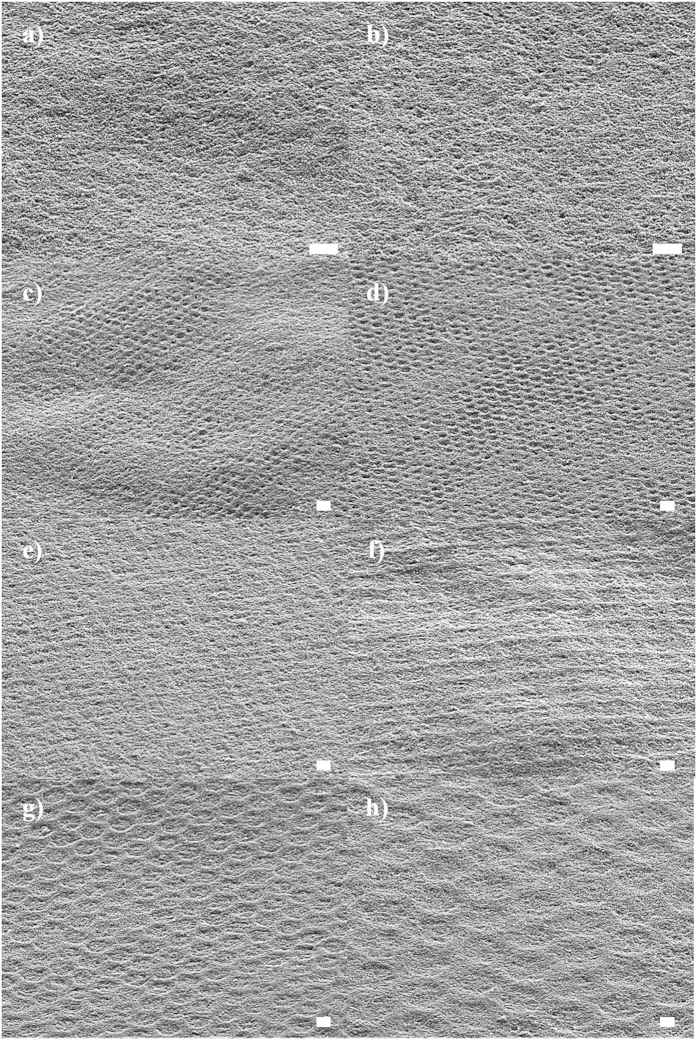
SEM images of patterned membranes coated with Pt/C catalyst (scale bars represent 10 μm). (a) Cir1, (b) Cir2, (c) Cir3, (d) Cir4, (e) Squ1, (f) Squ2, (g) Hex1, and (h) Hex2.

To evaluate the electrochemical properties and performance of the MEAs fabricated with the membranes patterned with various shapes and sizes, both sides of the synthesized membranes were directly coated with a Pt/C catalyst by a spraying method. The morphologies of the membranes covered with the Pt/C catalysis on the cathode side were also characterized with SEM and AFM. Fig. S2 shows cross-sectional images of the membrane/catalyst layer interface of each prepared MEA with four different patterned membranes (plain, circle, square and hexagon). The catalyst layers were strongly attached to the membrane surface without destruction of the original pattern structure. Interestingly, the random formation of macropores between the surface and patterns was observed, especially for the square and hexagon patterned membranes (indicated by a red circle), despite deep coverage by the catalyst layer. This feature could provide a better mass transfer of the reactant in the cathode for vigorous catalytic reactions. Remarkably, from the SEM images of Fig. 3, most of the catalyst-coated membranes clearly maintained the original pattern shape. However, Cir1 and Cir2 showed faint features of the patterns, which may be due to the small pattern size allowing the pores of the patterns to be completely covered with Pt/C catalyst. However, when we characterized the coated membranes using the AFM surface image, as shown in Fig. S3, none of the original pattern shapes were significantly perturbed, even for the circular pattern. This can be advantageous for a single MEA due to improvement in the 3-dimensional electrode structure. Furthermore, the protrusion of the pattern walls on the cathode side increased the area of intersection with the catalyst layer, resulting in increases in membrane surface area, catalytic activity, and electron/proton transfer pathways^12^,^13^,^14^,^15^,^16^,^17^,^18^,^19^. Therefore, to investigate the effect of this enhanced electrode structure, MEAs composed of the patterned membranes were prepared through the assembly of a catalyst-coated membrane, gas diffusion media, and Teflon gaskets without a pressing process.

### PEMFC performances

**Figure 4:**
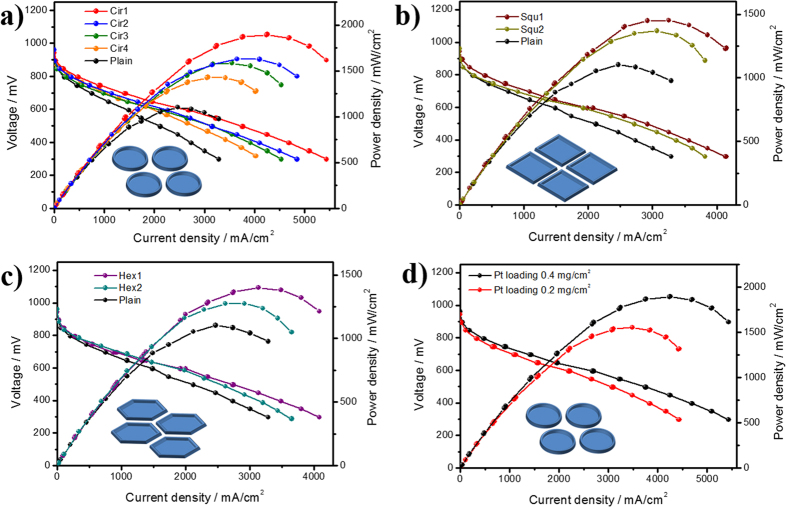
MEA performances of patterned and plain membranes. (a) Circle for Cir1, Cir2, Cir3, Cir4, (b) Square for Squ1, Squ2, (c) Hexagon for Hex1, Hex2 and (d) Comparison with the reduction of the Pt loading to 0.2 mg/cm^2^ for the Cir1.

To assess the PEMFC performances of the MEAs fabricated with variously patterned and unpatterned Nafion membranes, the polarization curves (I–V) for each MEA were measured at 75 °C as shown in Fig. 4. The performance parameters, such as open circuit voltages (OCVs), current densities (at 0.8 V, 0.6 V and 0.3 V) and maximum power densities, are listed in Table 1. The PEMFC performances of the MEAs with different patterns/sizes on the Nafion membrane are represented in three graphs (Fig. 4a–c) in which the power density (I-W) for the membranes with patterns of circles, squares and hexagons, respectively, were plotted to clearly compare the effect of the size of each pattern. All performance plots exhibited the typical shape of I-V and I-W curves, indicating that the PEMFC experiments were carefully performed. As listed in Table 1, the open-circuit voltage of all MEAs was nearly the same, with a range of 0.96–0.97 V without any gas crossovers. A small variance in OCV was presumably due to the small difference in the thickness of the patterned membranes, but the conductivity was nearly the same for all membranes. All the tested MEAs fabricated with the patterned membranes demonstrated greater performance than the plain membrane, regardless of the shape and size where the membrane surface area is considered the most dominant factor in designing high-performance MEAs for PEMFC.

Patterned membrane performance was further analysed to elucidate the complex behaviours of MEAs with different shapes and sizes. When evaluating the PEMFC performance of the prepared MEAs, the I–V curves can be examined in three different regions: the kinetic region (OCV–0.75 V), the ohmic polarization region (0.75–0.40 V), and the mass-transfer-controlled region (0.40–0.20 V)^18^. Herein, we focused on the potentials of 0.8, 0.6 and 0.3 V for each region, respectively, and the results are presented in Table 1. The region at 0.8 V was primarily influenced by the catalyst reaction of the Pt/C that is related to the kinetics of the chemical reactions of the MEAs with patterned membranes. Patterned membranes with various shapes and sizes improved the kinetics with incremental changes that were proportional to the increase in the membrane specific surface area. This result indicates that the activation reaction of Pt/C on the cathode side was generated by the enhancement in the oxygen-reduction reaction in the wider cathode catalyst region, resulting in improved PEMFC performance via an effective catalytic reaction^20^,^21^,^22^,^23^,^24^,^25^,^26^.

In addition to a catalytic reaction, mass transfer is another crucial for MEA performance. The limiting current density at low voltage, such as at 0.3 V of the mass-transfer-controlled region (0.40–0.20 V), is limited by the transport of reactant gas for the catalytic reactions in the cathode and illustrates the effectiveness of mass transfer of the reactant^17^,^18^ Generally, this region reflects performance associated with the interfacial design of the MEA. The mass transfer using patterned membranes was enhanced, and the incremental change was proportional to the membrane surface area, a similar tendency to the kinetic region. The highest limiting current density of 5434 mA/cm^2^ was obtained upon employing the membrane patterned with a 2-μm-diameter circle (Cir1), which is the smallest size of the circle pattern with the highest specific membrane surface area (1.698). Interestingly, the Squ1 membrane displayed greater electrochemical properties at a higher current density of 0.3 V than did the Cir4, despite the smaller membrane surface area (1.213) of the former compared to the latter (1.244). This indicates the importance of pattern shape and size. This result is further supported by the macropores produced at the corners of patterns with an angled structure, such as square and hexagon, as observed in the cross-sectional images of the MEA (Fig. S2). The presence of macropores improves mass transfer, leading to a higher MEA performance.

The increased performance of the patterned MEAs was primarily due to the improved interfacial properties at the catalyst/ionomer/membrane. This is basically due to the increase in the triple boundary phase properties is included in the ohmic polarization region (mid-current range), which represents resistance and good contact among the catalyst, Nafion binders, and the patterned Nafion membrane for an improved catalytic activity and mass transport of the reactant^18^. As shown in Table 1, the current densities of the patterned membrane-based MEAs at 0.6 V were 2597 (Cirl1), 2215 (Cirl2), 2068 (Cirl3), 1962 (Cirl4), 2077 (Squ1), 1921 (Squ2), 1935 (Hex1), and 1829 (Hex2) mA/cm^2^, which is consistent with the results observed for the performance at 0.8 and 0.3 V. All the performances are higher than the plain one and even higher than the commercial Nafion 212. The polarization curves for all the patterned membranes demonstrate dramatic improvement in the hydrogen electro-oxidation activity and mass transfer for the triple boundary phase in the cathode as compared to the plain Nafion membrane. These results shows even higher value than the line-patterned membranes from the previous work, indicating that the membrane surface area is an key factor for the MEA performance^32^. The highest current density obtained was for the membrane patterned with a circular pattern of 2 μm on the cathode side at 0.6 V (2597 mA/cm^2^); this was approximately 64% greater than that of the plain membrane (1488 mA/cm^2^ at 0.6 V). The maximum power density of the Cir1-base MEA reached 1906 mW/cm^2^, which is one of the highest findings in PEMFCs and even comparable with the DOE 2020 targets^29^.

### Reduction of the total Pt loading

One of the major hindrances limiting PEMFC commercialization is the amount of catalyst associated with Pt, whose high price represents approximately 30–40% of the total cost of the fuel cell^1^,^29^,^30^,^31^,^32^,^33^. The aim of this experiment was to investigate the effect of patterns at a lower catalyst loading. Figure S4 shows electrochemical analysis of different Pt loadings of 0.4 and 0.2 mg/cm^2^ deposited on the cathode with the patterned membrane Cirl1. As shown in Fig. S4a, the area of the CV curves decreased by approximately 35% when the catalyst loading was reduced to 50%. However, there was no significant change in the charge transfer resistance, as presented in Fig. S4b. From these properties, Fig. 4d shows that the performance of the resulting MEA did not decrease by two-fold, despite the same reduction in Pt loading. This result may be due to ohmic polarization, including the higher Pt utilization efficiency and better mass transfer due to the patterned structure on the cathode. The maximum power density for the loading of 0.2 mg/cm^2^ was 1555 mW/cm^2^. The DOE targets and commercial PEMFCs are based on normal air as an oxidizer, and thus the MEA with patterned membrane was also tested under air condition with the different amounts of Pt/C. As shown in Fig. S4c,d, the same tendency was obtained for the CV and impedance results. Interestingly, almost the same PEMFC performances with a high value (Fig. S4e) were achieved even at lower loading of 0.2 mg/cm^2^. As seen in Fig. S4f, our performance was comparable to the DOE current status for PEMFC applications, even though the MEA components and operation conditions were different from each other, e.g. no back pressure operations^29^. As a result, our methodology could be translated to the Global 2020 target of 0.125 mg/cm^2^ PGM loading, additionally with a catalyst development for further work.

## Discussion

### Electrochemical analysis

**Figure 5:**
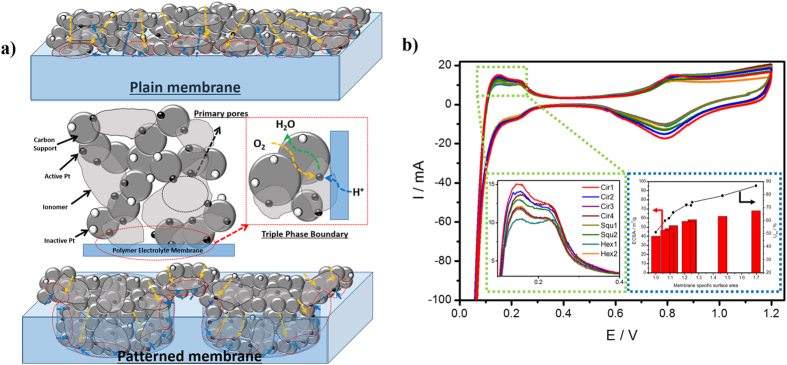
(**a**) Illustrations of the catalytic reaction and dominant pathways at the cathode of the MEA for the plain and patterned membranes, and (**b**) CV analysis of patterned membranes: Circle for Cir1, Cir2, Cir3, Cir4, Square for Squ1, Squ2, and Hexagon for Hex1, Hex2. The left inset shows the magnified plots of CV, and the right inset shows the ECSA change with specific surface area of the membranes.

To achieve a high PEMFC performance, it is important to design an effective cathode structure in order to maximize the triple boundary phase, which acts as the active sites for combination of O_2_ molecules, electrons and protons, as illustrated in Fig. 5a^16^^–23^. Based on this, Fig. 5a revealed that the patterned membranes may help to enhance the electrochemical properties of the catalyst layer due to the higher roughness of the membranes and shorter transport pathways of the reactants, resulting a production of more triple boundary phases from a thin catalyst electrode on the membrane surface^22^,^26^.

These expected electrochemical properties of the catalyst layer in the MEAs were characterized by CV measurements, as shown in Fig. 5b. The CV plots showed typical curves including strong hydrogen sorption/desorption peaks without any vertical shifts, irrespective of the patterned structure^20^,^21^,^22^,^23^,^24^,^25^. To evaluate the catalytic property of the catalysts layer, the hydrogen adsorption peak was primarily considered. As exposed in the magnified plots (the inset of Fig. 5b), the intensity of the hydrogen adsorption peak gradually increased with the patterned structure, and the area of the curves were always much greater than that of the plain Nafion membrane without a pattern. This result provides strong evidence for the enhanced catalytic property of the cathode due to patterned structures. Another important factor for the electrochemical properties of MEAs is the ECSA and Pt utilization (U_Pt_) of the catalyst Pt nanoparticles at the exterior electrode surface. It is crucial to investigate how the ECSA changes with the pattern structure since the oxygen reduction reaction in the cathode can be promoted by the wider catalyst reaction region^17^,^18^,^19^. Using the CV analysis and Equation S(1), the ECSA value was calculated using the electrical charge of H^ + ^ions from the hydrogen adsorption charge on the smooth Pt surface by integrating the hydrogen adsorption peak from the CV profile and subtraction of the estimated double layer charging current. As shown in the inset of Fig. 5 and Table S1, the ECSA ranged from 46.95 to 67.86 m^2^/g for the MEAs with patterned membranes, while the highest ECSA was achieved for the Cir1 with the highest membrane surface area. All the ECSA values are much greater than that of the plain membrane (40.12 m^2^/g) and followed the order of modified membrane surface area, indicating that surface area is the most important for increasing ECSA value. Higher ECSA was also expected to lead to high Pt utilization, which is another important factor for an effective triple phase boundary at the cathode interface.

U_Pt_ is considered as an another good indicator of the quality of the catalyst layer in an MEA as it reflects particle location, particle size, and size distribution^23^,^24^,^25^,^26^,^27^,^28^. It is possible to calculate the U_Pt_ in order to understand the catalytic behaviour and interface conditions in MEAs. The utilization of Pt can be attained from the CV measurements, accounting for the Pt particle size because electrochemical reactions occur at the effective surface area of the Pt particles (S_Pt_). Using Scherrer’s equation with a shape constant (K) of 0.9 on the Pt (111) diffraction peak from XRD patterns (Fig. S5), the mean particle sizes for all prepared MEAs were typically 3–4 nm. Meanwhile, a slight difference in XRD peak intensity and/or location between the patterned and plain membranes was detected, which is presumably due to the change in diffraction angle through the rough surface of the catalyst layers by spraying the catalyst ink directly onto the patterned membrane surface. The average particle size of 3.58 nm was obtain from the list in Table S1 in order to calculate the S_Pt_ values using the Equation S(2). Finally, the U_Pt_ values were calculated from the ratio of ECSA to the calculated S_Pt_, which expresses the amount of active surface Pt atoms for electrochemical reactions, as shown in Equation S(3). As summarized in Table S1, the U_Pt_ values for the circular, square and hexagonal MEAs were 86.64% (Cirl1), 79.12% (Cirl2), 74.12% (Cirl3), 71.63% (Cirl4), 72.04% (Squ1), 66.18% (Squ2), 61.78% (Hex1), and 59.95% (Hex2). All these values were greater than that of the plain membrane without a pattern (51.07%), indicating that the patterned structure plays an important role in improving the utilization of Pt catalyst. As shown in the inset of Fig. 5b, the utilization of Pt increased with the ECSA value, as the latter parameter was proportionally related to the specific surface area of the membrane. The introduction of the patterned structure to plain Nafion membranes enhanced the catalytic activity in the cathode and the electrochemical performance of MEAs due to the efficient distribution of Pt nanoparticles in the active contact zone for larger amount of triple boundary phase from the high Pt utilization, improving the kinetic region at 0.8 V, as presented in Fig. 4 and Table 1.

**Figure 6:**
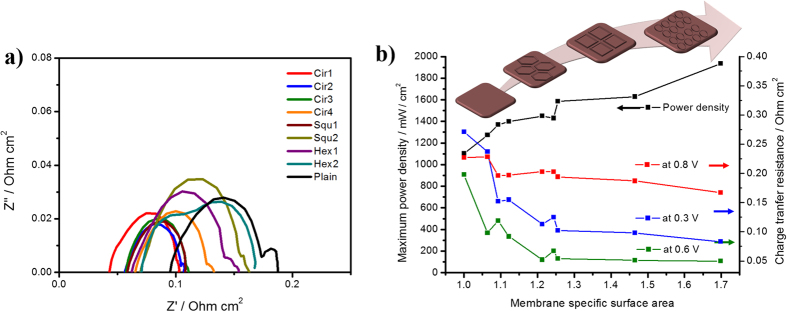
(**a**) Impedance analysis of MEAs with patterned and plain membranes at 0.6 V (inset: magnified results): Circle for Cir1, Cir2, Cir3, Cir4, Square for Squ1, Squ2, and Hexagon for Hex1, Hex2. (**b**) maximum power density and charge transfer resistance of MEAs with patterned and plain membranes at various voltages (0.8, 0.6 and 0.3 V) as a function of membrane specific surface area.

To further investigate the ohmic resistance and charge transfer resistance at the electrode/membrane of the MEAs, the Nyquist plots were measured at various potentials (0.8, 0.6 and 0.3 V) using EIS analysis, as presented in Fig. 6, Fig. S6 and Table S1. Generally, the ohmic loss, the x-axis intercept at the high frequency, is primarily associated with the membrane thickness and the proton transport resistance in both the membrane and cathode catalytic layer^34^,^35^. As shown in Fig. 6a, the intercept with the x-axis at a high frequency tended to shift to the right for the patterned membranes, indicating a decrease in ohmic resistance at 0.6 V. According to the summarized results in Table S1, the ohmic resistances of the patterned membranes at 0.6 V decreased by more than twice from 0.072 to 0.043 Ω cm^2^, while the plain membrane had a value of 0.096 Ω cm^2^. The decrease in ohmic resistance arises from the increases in roughness and membrane surface area, which is consistent with results of previous works of a line patterned membrane^32^. The proton transport resistance in the catalyst layer may be another reason, and the deviation in ohmic resistance was greater at the mass transfer region of 0.3 V (Table S1). Therefore, the pattern structure enhances proton and reactants transport when using patterns with shorter distances to the catalyst layer (Fig. 5a).

A similar tendency was observed for the charge transfer resistance results. From the EIS analysis, the charge transfer resistance at the electrode–membrane interface was obtained from the intercept with the x-axis at a low frequency, which is closely related to the electrode resistance due to the oxygen reduction reaction and transport property of the catalyst layer^36^,^37^. The charge transfer resistances were quantified from Fig. 6b and S6, and the values are summarized in Table S1. As previously discussed, the MEA performance increased with an increase in membrane specific surface area. Figure 6b demonstrates the relationship between MEA performance and behaviour of the charge transfer at different voltages (0.8, 0.6 and 0.3 V) according to membrane surface area. The charge transfer resistance at 0.6 V, which is responsible for the total cell resistance, decreased to a range of 0.111 to 0.050 Ω cm^2^ with increases in membrane surface area and power density. This decrease can be explained by the behaviour of the charge transfer resistance at the kinetic and mass transfer region, as shown in Fig. 6b. The resistance at 0.8 V was greater than that at 0.3 V, indicating that the resistance in the kinetic region was most dominant for the total charge transfer resistance of the electrode. This value further decreased with membrane surface area, reflecting the improved catalytic activity in the wider cathode catalyst region in which the oxygen-reduction reaction occurs. Remarkably, demonstrating a similar trend, the charge transfer resistance at 0.3 V for the mass transfer region also decreased with an increase in membrane surface area, but it showed a much larger deviation of 48.8% (from 0.241 to 0.083 Ω cm^2^) than the 15.2% charge transfer difference at 0.8 V (from 0.227 to 0.167 Ω cm^2^). The decreased cell resistance was primarily due to the contribution of the catalyst layer resistance. The mass transfer region was more influenced by the pattern structure of the membrane, especially with the angled shape as confirmed before (Fig. S2). By using the patterned structure as illustrated in Fig. 5a, shorter proton and electron pathways through the 3-dimensional pore structures in the cathode were produced, which resulted in an effective mass transfer of both reactants and products and reduced the electrode resistance. Based on these results, a patterned structure on the membrane improves the electrochemical property and performance of PEMFCs.

### Operation at low relative humidity conditions

One advantage of the patterned membrane is the possibility of use in low relative humidity (RH) conditions due to the effective MEA structure for better mass transfer, which will be a practically important factor for operating a fuel cell in severe conditions^12^,^13^. When the MEA is operated in dehydration condition, a low ionic conductivity hinders the access of protons to the catalyst surface, reducing the actual number of reactive active sites in the catalyst layer, thus increasing the activation polarization^38^,^39^,^40^. Figure S7 displays the comparison of the electrochemical properties, including I-V, CV curves and impedance analysis, between the patterned and plain membranes at low RH conditions. All of the results are summarized in Table S2. As shown in the I-V curves in Fig. S7a, both MEAs were tested at RH conditions of 100, 75 and 50% to investigate different behaviours. The initial performance was much greater than that of the plain membrane-based MEA, and a smaller performance drop was achieved for the patterned membrane, which experienced a 47.8% decrease compared to the 68.4% for the plain membrane when the RH changed from 100% to 50%. In particular, the electrochemical property at the kinetic region did not decrease significantly, whereas a large decrease was observed at the mass transfer region. This is also supported by CV and impedance analyses. The CV curves in Fig. S7b were not significantly degraded at the RH of 50% for either tested MEA, though there was a small decrease in ECSA (Table S2). Alternatively, large changes in membrane resistance and charge transfer resistance were observed, as exposed in Fig. S7c. In general, both resistances increased at dehydration conditions; however, the patterned membrane showed a smaller drop in resistance, especially the charge transfer resistance, indicating that the patterned structure enhances the mass transport to a wide cathode catalyst region under the dehydrated condition.

In summary, MEAs based on the patterned membrane were fabricated through a simple method using PDMS molds with different shapes and sizes in order to improve the interface and electrochemical properties of the membrane/catalyst layer. In particular, we investigated the effect of pattern shape and size on Pt utilization according to the membrane surface area in order to elucidate the effect of pattern structure on the catalytic reaction. Greater than 80% Pt utilization was achieved by the membrane with a circular pattern of 2 μm, which has the highest surface area. Even though the size of the patterns was the primary factor in enhancing the contact at the membrane/electrode interface, an interesting behaviour of the chemical properties was observed among the different shaped patterns. This approach could enhance the electrochemical activity on the cathode side, resulting in a huge increase in PEMFC performance, i.e., power densities of 1906 mW/cm^2^ and 1555 mW/cm^2^ at Pt loadings of 0.4 and 0.2 mg/cm^2^, respectively, may represent a possibility to overcome the 2020 DOE target. Furthermore, our study suggests that patterned membranes are highly active and effective structures for improving mass transfer and catalytic properties, which are very useful for PEMFC operation at low RH conditions and could reduce the amount of catalyst required and targeted by DOE, thereby dramatically reducing the cost of the fuel cell stack.

## Experimental Procedures

A 184 silicon elastomer and curing agent were purchased from Dow Corning. A Nafion perfluorinated resin solution (5 wt% in lower aliphatic alcohols and water, contains 15–20% water) was purchased from Aldrich. All the chemicals and solvent were used without extra purification.

First, for patterned PDMS mold fabrication, a patterned silicon master was prepared via a photo-etching process with the mask contacting various patterns, i.e., circles, hexagons and squares. After it was placed into the square dish, a few drops of tridecauoro-1,1,2,2-tetrahydrooctyl-1-trichlorosilane (TFPCS) were cast onto the master, which was stored in a vacuum oven at RT for 30 min. The PDMS solution was prepared by mixing the 184 silicon elastomer and the curing agent at 10:1 (weight ratio), and the mixture was poured onto the silicon master in the square dish. After storing in air for degassing, the membranes were stored in an oven at 70 °C for 1 h. Finally, the cured, patterned PDMS mold was carefully detached from the silicon master. For fabrication of the patterned Nafion membrane, a specific amount of the Nafion solution was poured into the PDMS mold and dried in a closed system at R.T. for 12 h. The thickness of the patterned Nafion membrane was maintained approximately 50 μm, and a few patterned membranes were prepared repeatedly as the PDMS mold was reusable. The bare and catalyst-deposited surface structures of the patterned Nafion membrane were characterized using a field-emission scanning electron microscope (FE-SEM, AURIGA, Carl Zeiss) and a non-contact atomic force microscope (AFM, XE-BIO, Park system).

The MEA fabrication was fixed for all prepared membranes using the catalyst-coated membranes (CCM) method. The commercial Pt/C catalyst (Johnson Matthey, 40 wt% Pt on carbon black) was directly sprayed onto both sides of the prepared membranes using an airbrush gun. The slurry used as catalyst ink for the spray contained 0.1 g Pt/C, 0.4 g de-ionized water, 0.8 g Nafion solution, and 1.2 g isopropyl alcohol (Aldrich). The catalyst inks were mechanically stirred and ultrasonicated to allow good mixing of the ionomer and Pt nanoparticles. Each step was repeated five times. The catalyst loadings were 0.4 and 0.2 mg/cm^2^ at an active surface area of 1 cm^2^. The catalyst-coated membranes were dried at 100 °C for 1 h to remove residual solvent. Finally, the MEAs were fabricated by assembly of the catalyst-coated membrane, gas diffusion media (SGL 10BC), and Teflon gasket without hot pressing. The electrochemical experiments were performed using single PEMFCs (Fuel Cell Technology, USA). The flow rates of pure hydrogen (H_2_) and oxygen/air (O_2_ or Air) were 0.2 and 0.3 L∙min^–1^ as the fuel and oxidation gas, respectively, with a stoichiometry of 1/1.5 (H_2_/O_2_ or Air). The standard electrochemical experiments were performed at 75 °C and 100% relative humidity (RH) without back pressure. To control the humidification condition, the feeding reactive gases were preheated at different temperatures using a humidifier. The polarization curves (current density vs. voltage) were determined using a fuel cell load of KFM2030 (KIKUSUI) in order to investigate the PEMFC performances. To analyze the electrochemical properties, *in situ* CV and EIS measurements were performed using an FC impedance meter (PGSTAT-30 Autolab). CV scans were performed in the range of 0 to 1.35 V at a scan rate of 50 mV/s to analyze the catalyst layer at the electrode. The membrane/electrode resistances were investigated using EIS analysis under constant flow rate by applying amplitude of 10 mV peak-to-peak over the frequency range of 100 mHz–10 kHz. The cell potentials were changed (*E*cell = 0.8, 0.6 and 0.3 V) for insight into the interfacial reactions and mass transport in the PEMFC.

## Additional Information

**How to cite this article**: Jeon, Y. *et al.* Interface-designed Membranes with Shape-controlled Patterns for High-performance Polymer Electrolyte Membrane Fuel Cells. *Sci. Rep.* **5**, 16394; doi: 10.1038/srep16394 (2015).

## Supplementary Material

Supplementary Information

## Figures and Tables

**Table 1:** Characteristics of the prepared membranes and MEA performances.

Samples	Diameter (um)^a^	Thickness (um)	Specific Membrane Surface Area	Open Circuit Voltage (mV)	Current density at 0.8 V (mA/cm^2^)	Current density at 0.6 V (mA/cm^2^)	Current density at 0.3 V (mA/cm^2^)	Maximum power density (mW/cm^2^)
Cir1	2	45	1.698	970	436	2597	5434	1906
Cir2	3	46	1.465	970	358	2215	4851	1629
Cir3	5.5	48	1.255	970	348	2068	4535	1584
Cir4	7	48	1.244	963	256	1962	4036	1430
Squ1	9	49	1.213	963	312	2077	4136	1450
Squ2	17	46	1.093	960	245	1921	3819	1369
Hex1	15	45	1.122	963	254	1935	4086	1398
Hex2	25	46	1.064	960	206	1829	3653	1273
Plain	0	45	1	963	187	1488	3287	1102
Commercial N212	—	50	1	970	191	1580	3280	1105

^a^Size means the diameter of circle, side of square, and distance between parallel sides of hexagon.
