# Supplementary material for: Interface-designed Membranes with Shape-controlled Patterns for High-performance Polymer Electrolyte Membrane Fuel Cells
Source: Sci Rep. 2015 Nov 10;5:16394. doi: 10.1038/srep16394 (PMC4639844; doi:10.1038/srep16394)
Supplement: Supplementary Information [file srep16394-s1.doc]

Supporting Information

Interface-designed Membranes with Shape-controlled Patterns for High-performance Polymer Electrolyte Membrane Fuel Cells

Yukwon Jeon^†1^, Dong Jun Kim^†1^, Jong Kwan Koh^1^, Yunseong Ji^1^, Jong Hak Kim^*1^, and Yong-Gun Shul^*1^

^1^ Department of Chemical and Biomolecular Engineering, Yonsei University, 50 Yonsei-ro, Seodaemun-gu, Seoul 120-749, Korea

† These authors contributed equally to this work.

* Corresponding authors: E-mail: jonghak@yonsei.ac.kr (Prof. J.H. Kim), and shulyg@yonsei.ac.kr (Prof. Y.G. Shul)

**Calculation of ECSA, S_Pt_, and U_Pt_**

Equation S(1) was used to calculate the ECSA (m^2^/g) values of the Pt/C electrocatalyst on the cathode side of the MEAs:^1-4^

$ECSA=\frac{100\cdot Q_{H}}{\Gamma\cdot L}$ (1)

Q_H_ is the electrooxidation charge of the adsorbed hydrogen on the Pt surface integrated from the hydrogen adsorption peak. The values are summarized in Table S1. Γ is the electrical charge associated with monolayer adsorption of the hydrogen on the Pt catalyst surface (generally 21 mC/cm^2^), and L is the mass of the Pt in the cathode (mg).

Assuming that each Pt nanoparticle had a uniform spherical shape, the surface area of Pt (S_Pt_) was calculated using Equation S(2):^3^

$S_{Pt}=\frac{surface}{mass}=\frac{\pi\cdot D^{2}}{\pi D^{3}/6}=\frac{6}{\rho\cdot D}$ (2)

ρ is the mass density of Pt (21.4 g/cm^3^), and D is the mean particle size of Pt calculated from XRD patterns (Figure S5) of each catalyst layer in the cathode.

Finally, we can calculate the U_Pt_ from the ratio of ECSA to the calculated S_Pt_, which expresses the amount of active surface Pt atoms for the electrochemical reactions, as shown in Equation S(3).^5-7^

$U_{Pt}=\frac{ECSA}{S_{Pt}}$ (3)

**Figure S1.** Photographs of diffraction patterns induced by a laser pointer: (a) Cir1, (b) Cir2, (c) Cir3, (d) Cir4, (e) Squ1, (f) Squ2, (g) Hex1, and (h) Hex2.


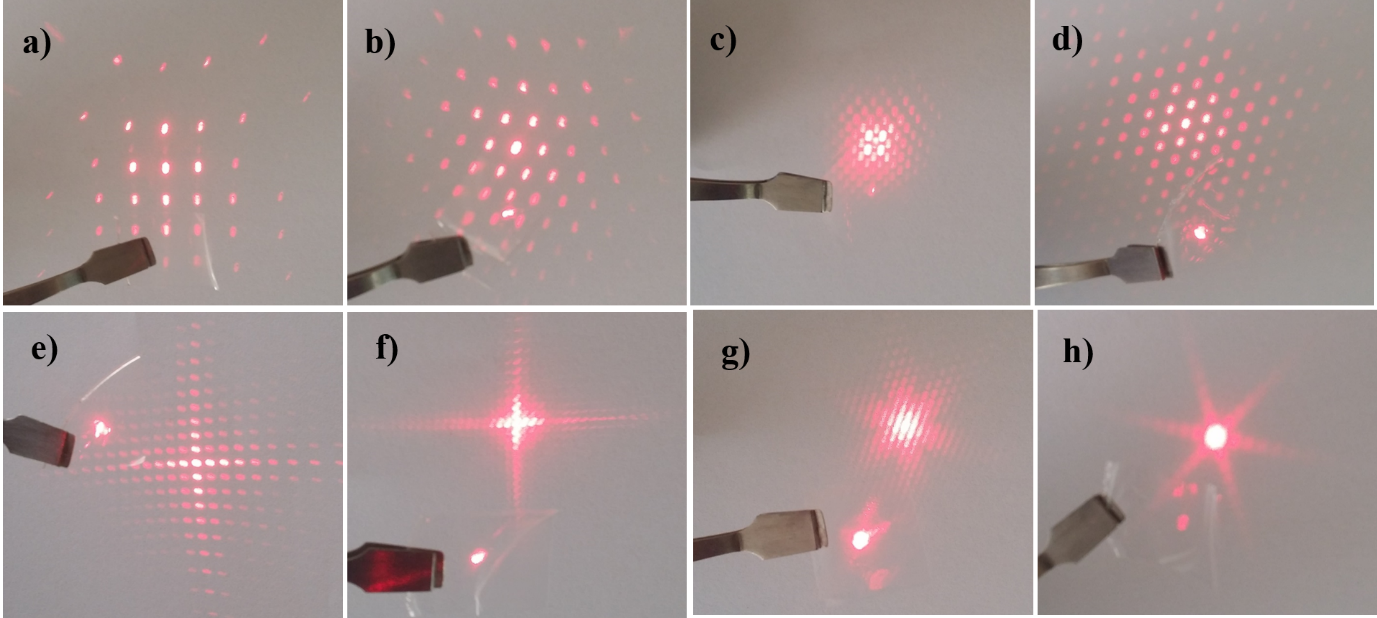


**Figure S2.** Cross-section of the patterned membranes coated with Pt/C catalyst (scale bars represent 500 nm): a) plain, b) circle, c) square, and d) hexagon.


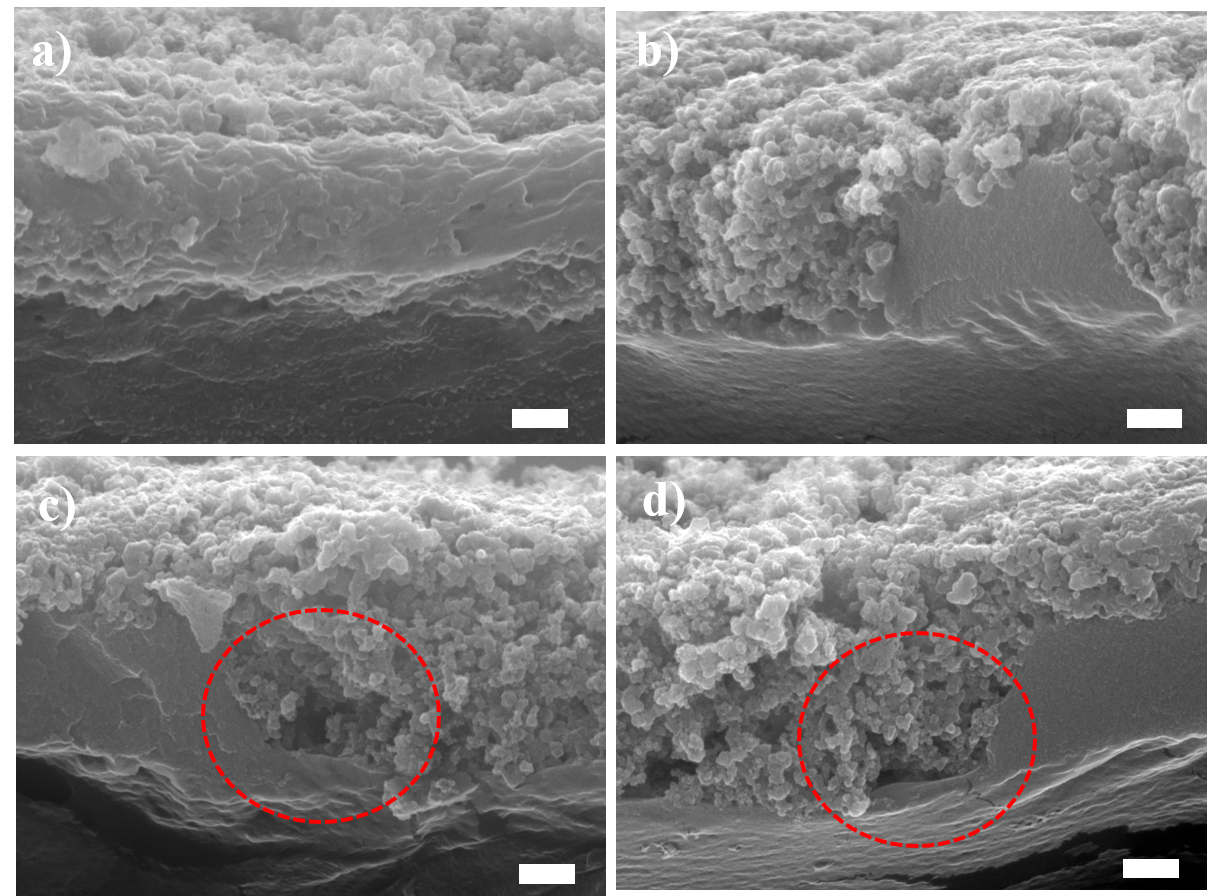


**Figure S3.** Three-dimensional and surface AFM images of patterned membranes coated with Pt/C catalyst: circle ((a), (b)), square ((c), (d)), and hexagon ((e), (f)).


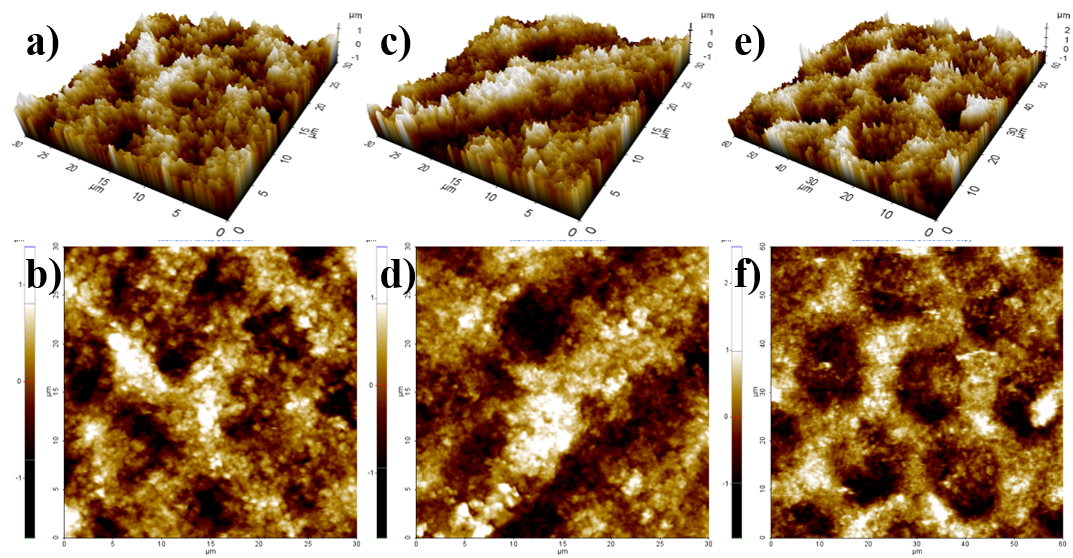


**Figure S4.** Different amounts of Pt loading for the single MEAs fabricated with patterned membranes: a) CV (oxygen), b) impedance (oxygen), c) CV (air), d) impedance (air), e) I-V measurments, and comparison of the power densities with the DOE reports.^8,9^ (^a^H_2_/O_2_, ^b^H_2_/Air (Cir1, Pt/C, 75 ^o^C, no back pressure), and ^c^PEM (3M, 24 μm), Pt_3_Ni_7_ based nano catalysts, 80 ^o^C, H_2_/Air, back pressure of 150/150 kPa)


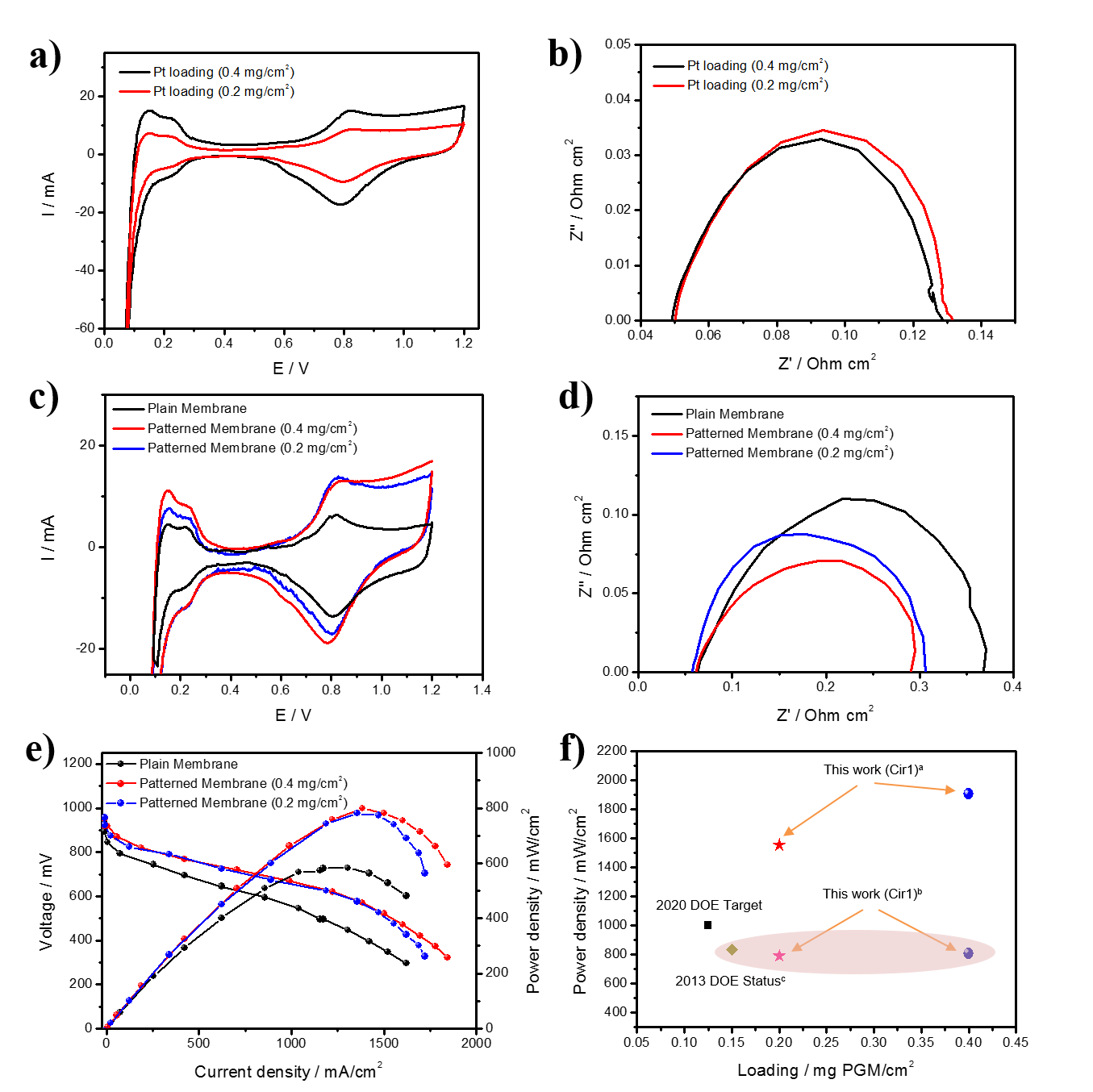


**Figure S5.** XRD of the catalyst layers sprayed onto the prepared membranes before the performance test.


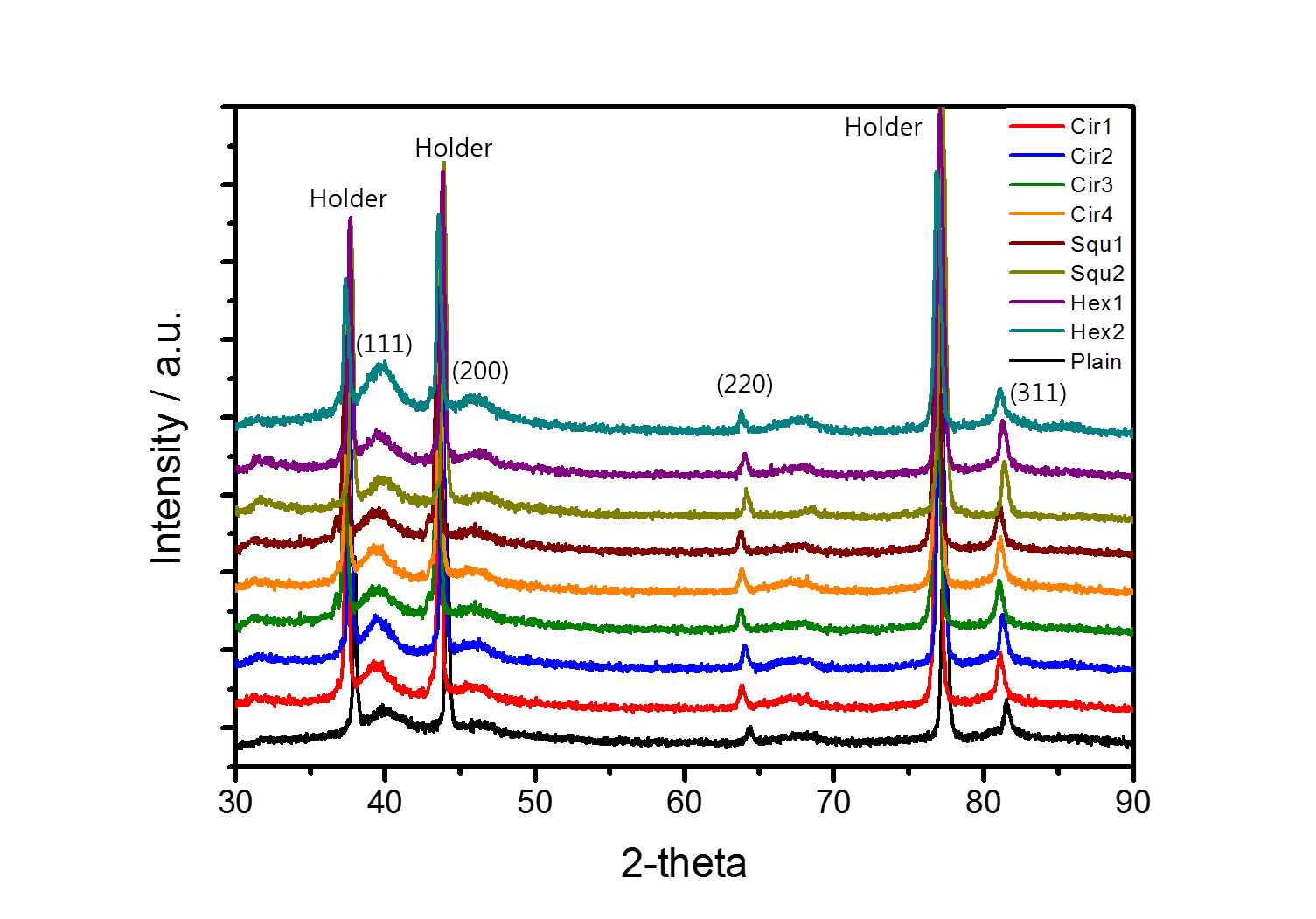


**Figure S6.** Nyquist plots of single MEAs fabricated with patterned membranes at voltages of a) 0.8 V and b) 0.3 V.


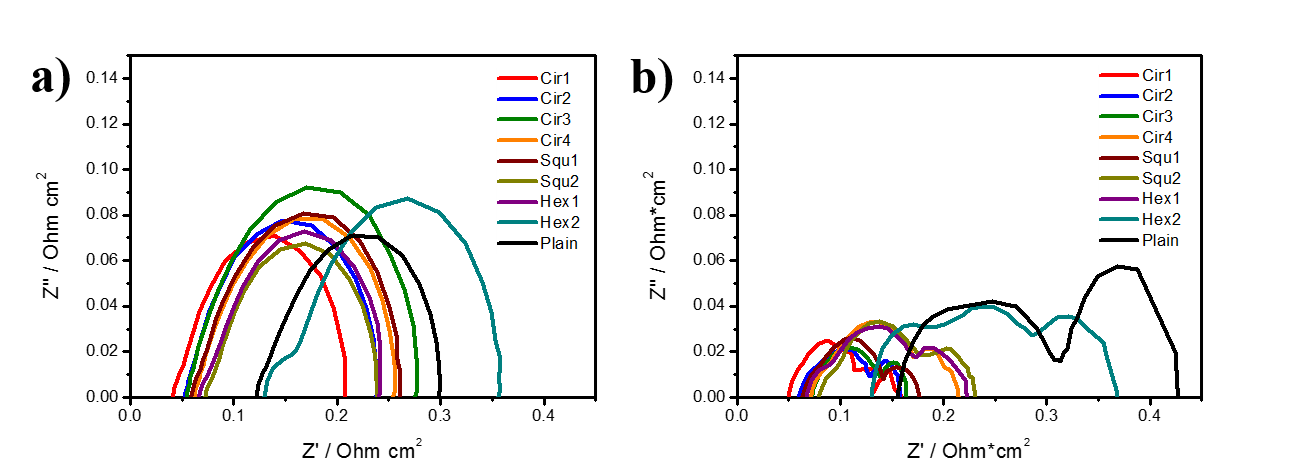


**Table S1.** Electrochemical properties of the prepared MEAs.

| Samples | Specific membrane surface area |  | Q_H_ [mC] | ECSA [m^2^/g] | Pt mean particle size  [nm] | U_Pt_ [%] |  | Ohmic resistance at 0.8V  [Ω cm^2^] | Ohmic resistance at 0.6V  [Ω cm^2^] | Ohmic resistance at 0.3V  [Ω cm^2^] | Charge transfer resistance at 0.8V  [Ω cm^2^] | Charge transfer resistance at 0.6V  [Ω cm^2^] | Charge transfer resistance at 0.3V  [Ω cm^2^] |
| --- | --- | --- | --- | --- | --- | --- | --- | --- | --- | --- | --- | --- | --- |
| Cir1 | 1.698 |  | 57.15 | 67.86 | 3.21 | 86.64 |  | 0.041 | 0.043 | 0.052 | 0.167 | 0.050 | 0.083 |
| Cir2 | 1.465 |  | 52.05 | 61.94 | 3.35 | 79.12 |  | 0.052 | 0.056 | 0.061 | 0.187 | 0.051 | 0.098 |
| Cir3 | 1.255 |  | 47.12 | 56.09 | 3.48 | 74.12 |  | 0.054 | 0.057 | 0.071 | 0.203 | 0.054 | 0.102 |
| Cir4 | 1.244 |  | 48.76 | 57.04 | 3.44 | 71.63 |  | 0.061 | 0.065 | 0.071 | 0.194 | 0.067 | 0.125 |
| Squ1 | 1.213 |  | 47.39 | 56.41 | 3.51 | 72.04 |  | 0.058 | 0.058 | 0.063 | 0.203 | 0.052 | 0.113 |
| Squ2 | 1.093 |  | 40.64 | 48.38 | 3.67 | 66.18 |  | 0.068 | 0.071 | 0.075 | 0.197 | 0.119 | 0.152 |
| Hex1 | 1.122 |  | 43.54 | 51.83 | 3.59 | 61.78 |  | 0.066 | 0.062 | 0.067 | 0.196 | 0.092 | 0.155 |
| Hex2 | 1.064 |  | 39.44 | 46.95 | 3.71 | 59.95 |  | 0.075 | 0.072 | 0.082 | 0.228 | 0.098 | 0.237 |
| Plain | 1.000 |  | 33.61 | 40.12 | 3.84 | 51.07 |  | 0.122 | 0.096 | 0.155 | 0.227 | 0.111 | 0.241 |

**Figure S7.** Electrochemical properties of single MEAs fabricated with patterned membranes at different RH conditions: a) I-V, b) CV and c) impedance analysis.


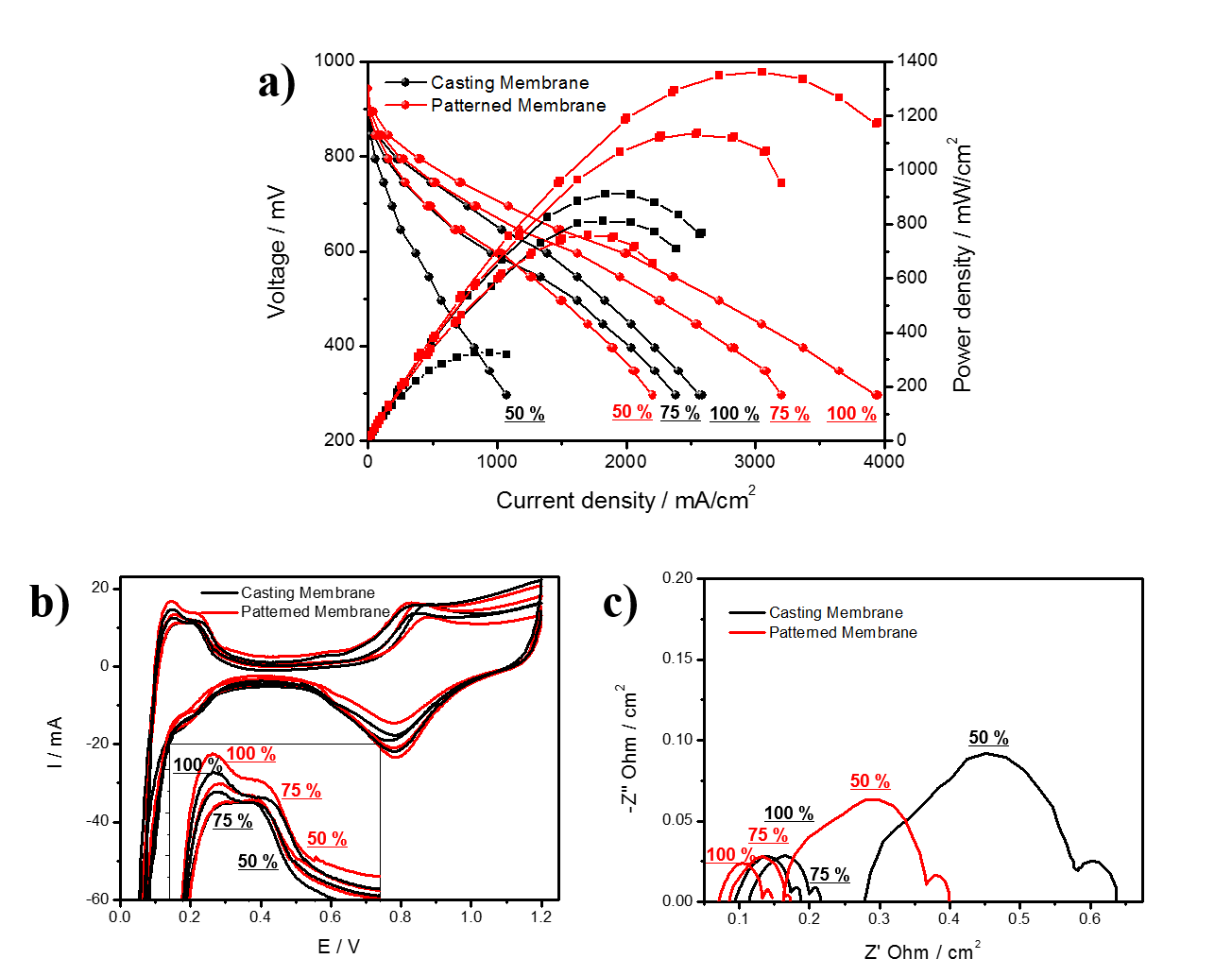


**Table S2.** Electrochemical properties of single MEAs fabricated with patterned membranes at different RH conditions.

| Plain Membrane | | | | | |  | Patterned Membrane | | | | | |
| --- | --- | --- | --- | --- | --- | --- | --- | --- | --- | --- | --- | --- |
| RH (%) | Current density at 0.6V  (mA/cm^2^) / (%) | | ECSA  (m^2^/g) | Membrane resistance  (Ω cm^2^) | Charge transfer resistance  (Ω cm^2^) |  | RH (%) | Current density at 0.6V  (mA/cm^2^) / (%) | | ECSA  (m^2^/g) | Membrane resistance  (Ω cm^2^) | Charge transfer resistance  (Ω cm^2^) |
| 100 | 1488 | 100 | 40.1 | 0.096 | 0.11 |  | 100 | 2068 | 100 | 58.0 | 0.065 | 0.067 |
| 75 | 955 | 64.8 | 31.2 | 0.114 | 0.122 |  | 75 | 1621 | 81.5 | 38.2 | 0.086 | 0.087 |
| 50 | 439 | 31.6 | 25.4 | 0.279 | 0.357 |  | 50 | 1038 | 52.2 | 27.3 | 0.162 | 0.237 |

**Reference**

1. Y. Qiao and C. M. Li, *J. Mater. Chem.* **21**, 4027–4036 (2011).

2. S. Chen, Z. Wei, H. Li and L. Li, *Chem. Comm.* **46**, 8782 (2010).

3. M. Uchida, et al., *Phys. Chem. Chem. Phys.* **15**, 11236–11247 (2013).

4. S. S. J. Aravind and S. Ramaprabhu, *ACS Appl. Mater. Inter.* **4**, 3805–3810 (2012).

5. D. Zhao and B.-Q. Xu, *Angew. Chem.* **118**, 5077–5081 (2006).

6. W. Zhu, et al., *Electrochimica Acta*, **55**, 2555–2560 (2010).

7. K. Karan, *Electrochem. Commun.* **9**, 747–753 (2007).

8. N. Jha, et al., *Sci. Rep.* **3** (2013).

9. U .S.DRIVE Partnership, *DOE Fuel Cell Technical Team Roadmap*, (2013).
